# Anatomical osteocartilaginous reconstruction of MacLaughlin lesion in chronic locked posterior shoulder dislocation: A novel technique

**DOI:** 10.1016/j.ijscr.2025.111116

**Published:** 2025-03-05

**Authors:** Ala Aloui, Jules Cavailhès, Remy Coulomb, Jeffrey Michaud, Pascal Kouyoumdjian, Olivier Mares

**Affiliations:** a University Hospital Center of Nîmes, 4 Professeur Robert Debré street, 30900 Nîmes, France; b Faculty of Medicine Montpellier-Nîmes, 186 Carreau de Lanes Road, 30900 Nîmes, France

**Keywords:** Posterior shoulder dislocation, Chronic, Surgery, Outcomes

## Abstract

**Introduction:**

Chronic Posterior shoulder dislocation presents significant challenges, with treatment decisions influenced by the duration of the injury and the severity of humeral head damage. This report highlights an innovative technique for reconstructing humeral head defects using an osteocartilaginous graft from costal cartilage, offering insights into the surgical procedure and the patient's favorable recovery outcomes.

**Presentation of case:**

The patient, a 23-year-old male with no significant medical history, was treated for a neglected posterior shoulder dislocation of two months' duration, complicated by a reverse Hill-Sachs lesion. The treatment involved an open reduction and reconstruction of the reverse Hill-Sachs lesion using costal cartilage.

At the six-month follow-up, the patient was evaluated and demonstrated excellent shoulder function, with a Constant score of 92.5.

**Discussion:**

This technique introduces costal cartilage grafting for reverse Hill-Sachs lesion reconstruction, offering a novel anatomical approach to restore humeral head integrity. Unlike traditional methods, it ensures excellent biocompatibility, joint congruence, and stability while preserving shoulder mobility. Costal cartilage is abundant, versatile, and widely used in reconstructive surgery, yet its application in posterior shoulder dislocation remains unexplored. This method avoids tendon transfer complications and hardware-related issues, providing a promising alternative with reduced long-term risks and improved functional outcomes.

**Conclusion:**

Our technique utilizing an osteocartilaginous graft harvested from the costal cartilage, combined with meticulous soft tissue management, demonstrated a promising approach to reconstruct the humeral head and restore shoulder stability for chronic posterior shoulder dislocation with large reverse Hill-Sachs lesion.

## Highlights

• Posterior shoulder dislocation is rare and often misdiagnosed clinically.
• A novel technique uses costal cartilage grafts to reconstruct humeral head defects.
• The approach restores shoulder stability and preserves joint functionality.
• The study details the surgical method and reports successful patient outcomes.

## 1 Introduction

Posterior shoulder dislocation is uncommon, representing approximately 2 %–4 % of all shoulder dislocations [1]. Mechanisms causing this injury include electric shock, epileptic seizures, or high-energy trauma [2], posing challenges for orthopedic surgeons. Acute posterior dislocations are often missed due to insufficient imaging or inadequate physical examination, unlike the more obvious symptoms of anterior dislocations. This type of dislocation can cause impression fractures on the front humeral head surface, called “MacLaughlin lesion” or “reverse Hill-Sachs lesion.” It results in significant symptoms, increasing risks of recurrent dislocations, joint damage, and early arthritis [3]. Although acute shoulder dislocation is well-documented, studies on chronic posterior dislocation remain limited, with no consensus on optimal treatment.

Managing neglected posterior glenohumeral dislocation requires an individualized approach. Treatment decisions depend on humeral head damage extent and dislocation duration. Options include conservative measures, like closed reduction and immobilization, or surgical interventions addressing humeral head defects through anatomical or non-anatomical reconstruction [4]. Anatomical procedures restore the humeral head's natural shape using open reduction with autograft or allograft materials [5]. Non-anatomical techniques enhance stability by filling the defect with the subscapularis tendon [6], first described by MacLaughlin.

This technical note introduces a novel anatomical technique to reconstruct humeral head defects using an osteocartilaginous graft from costal cartilage, combined with soft tissue surgical interventions. This study details the technique and reports patient progress and outcomes.

This work has been reported in line with the SCARE criteria [7] and it has been reported in line with the PROCESS criteria [8].

## 2 Materials and methods

### 2.1 Case presentation

**Fig. 1:**
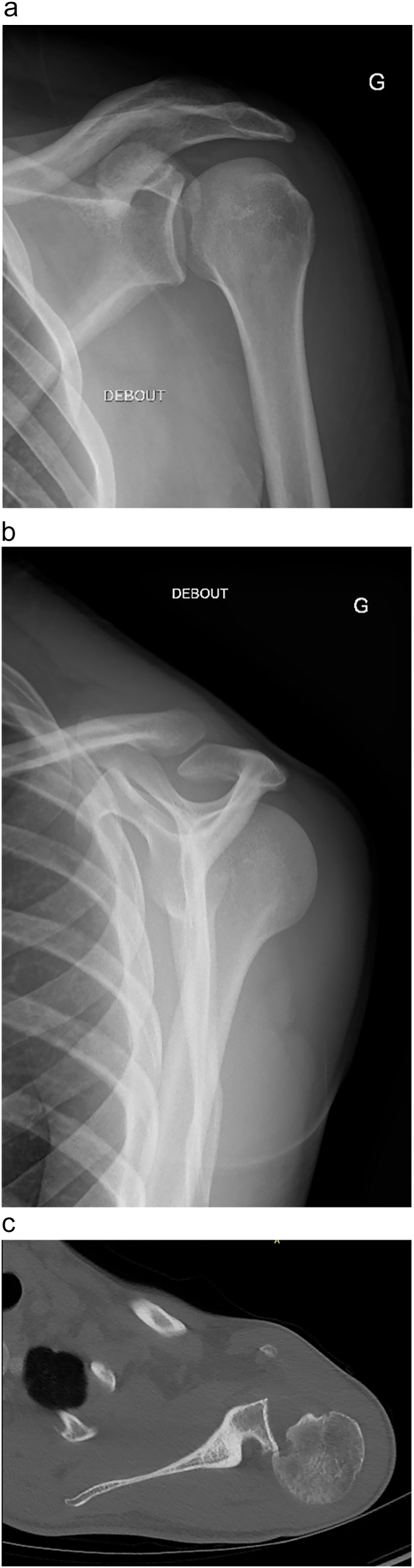
a, b: X-rays showing left shoulder posterior dislocation. c: CT scan showing Posterior left shoulder dislocation with reverse hill Sachs lesion.

Our patient was a 23-year-old right-handed man with no significant medical history. The initial trauma occurred during a road accident, involving a severe anteroposterior impact on the patient's left shoulder. He was assessed in the emergency room on the day of the accident; however, a posterior dislocation of his left shoulder was unfortunately missed during the initial evaluation. The patient received medical treatment and was immobilized using the Mayo clinic method. Two months later, due to a lack of improvement, characterized by persistent left shoulder pain and significant limitation of motion, he sought additional medical consultation. Physical examination revealed a marked restriction in both active and passive range of motion in the left shoulder, particularly in external rotation. Plain radiographs and a CT scan confirmed a neglected posterior dislocation of the left shoulder, accompanied by a reverse Hill-Sachs lesion involving more than one-third of the humeral head circumference (Fig. 1). The medical decision was to perform an open reduction with reconstruction of the humeral head defect.

### 2.2 Surgical technique

**Fig. 2:**
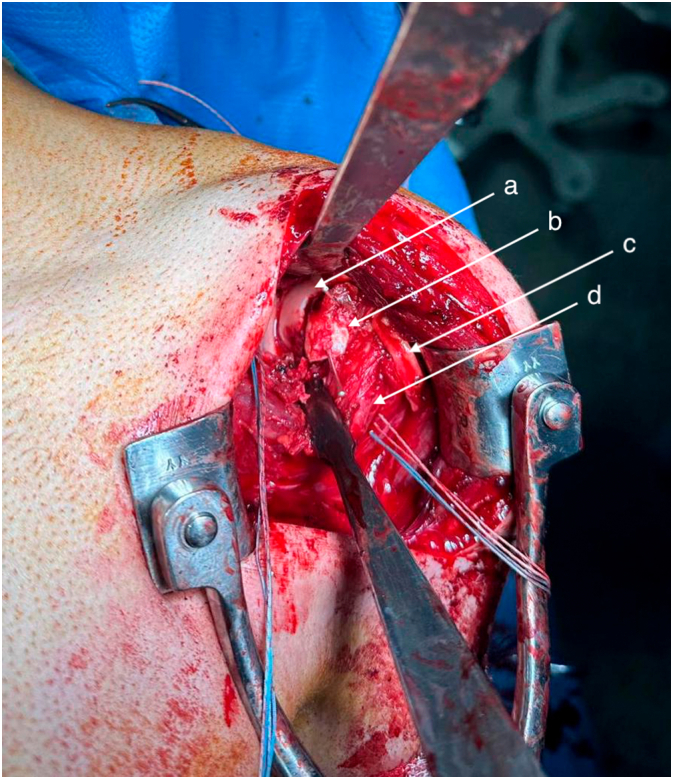
Intraoperative view of reverse Hill-Sachs lesion. a - Humeral head. b - Chondrocostal graft fixed in reverse Hill-Sachs lesion. c - Long head of the biceps. d - Subscapularis muscle.

The patient was installed in a beach chair position. A standard deltopectoral approach was performed, and the subscapularis was preserved since adequate access was achieved through the rotator interval. A 2 cm by 3 cm defect was identified on the anteromedial surface of the humeral head, caused by impingement against the posterior glenoid rim. The defect was thoroughly debrided to reveal a clean bony bed, which was prepared for grafting (Fig. 2).

The costal cartilage was chosen as the graft for our patient. The incision was made just medial to the 10th costochondral junction. The 10th rib was chosen based on our department's preference as we believe it results in a less visible scar. A deep incision was extended through the skin and subcutaneous fat to expose the underlying fascia. Retractors were used to ensure a clear surgical view, and a longitudinal incision was made between the external oblique muscle and the rectus abdominis muscle. The muscles were carefully dissected parallel to their fibers. Underneath a layer of loose areolar tissue, the costal cartilage was exposed. The cartilage was meticulously separated from the perichondrium, which was incised to match the required graft length.

A split costal graft measuring approximately 1 cm in height, 3 cm in length, and 1 cm in thickness was harvested, ensuring the deep perichondrium and the remaining costal cartilage remained intact. The lateral and inferior aspects of the cartilage were preserved, and the perichondrium was sutured over the gap left by the graft.

**Fig. 3:**
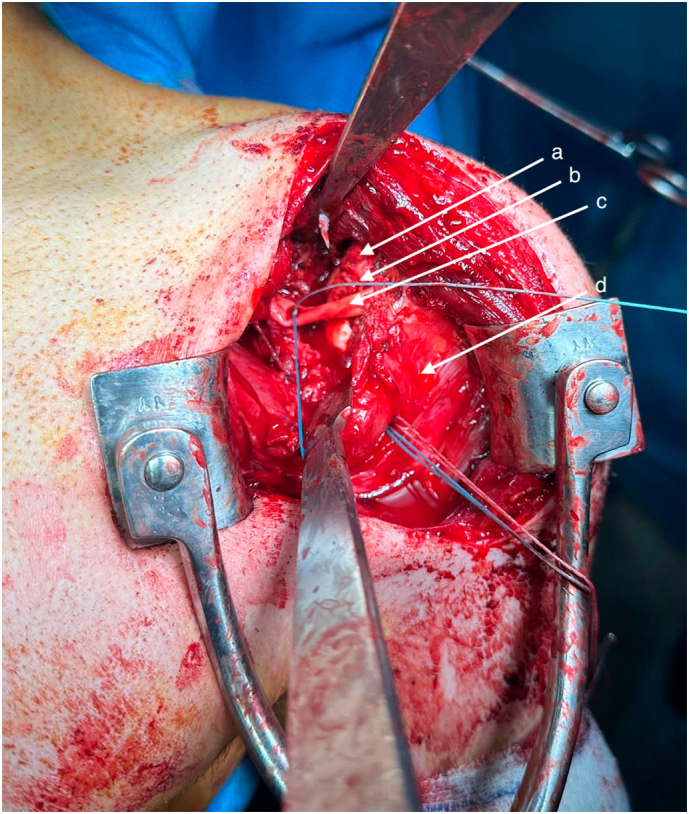
Intraoperative view of chondrocostal graft and soft tissue repair. a - Humeral head. b - Costal cartilage graft fixed in reverse Hill-Sachs lesion. c - Long head of the biceps. d - Subscapularis muscle.

**Fig. 4:**
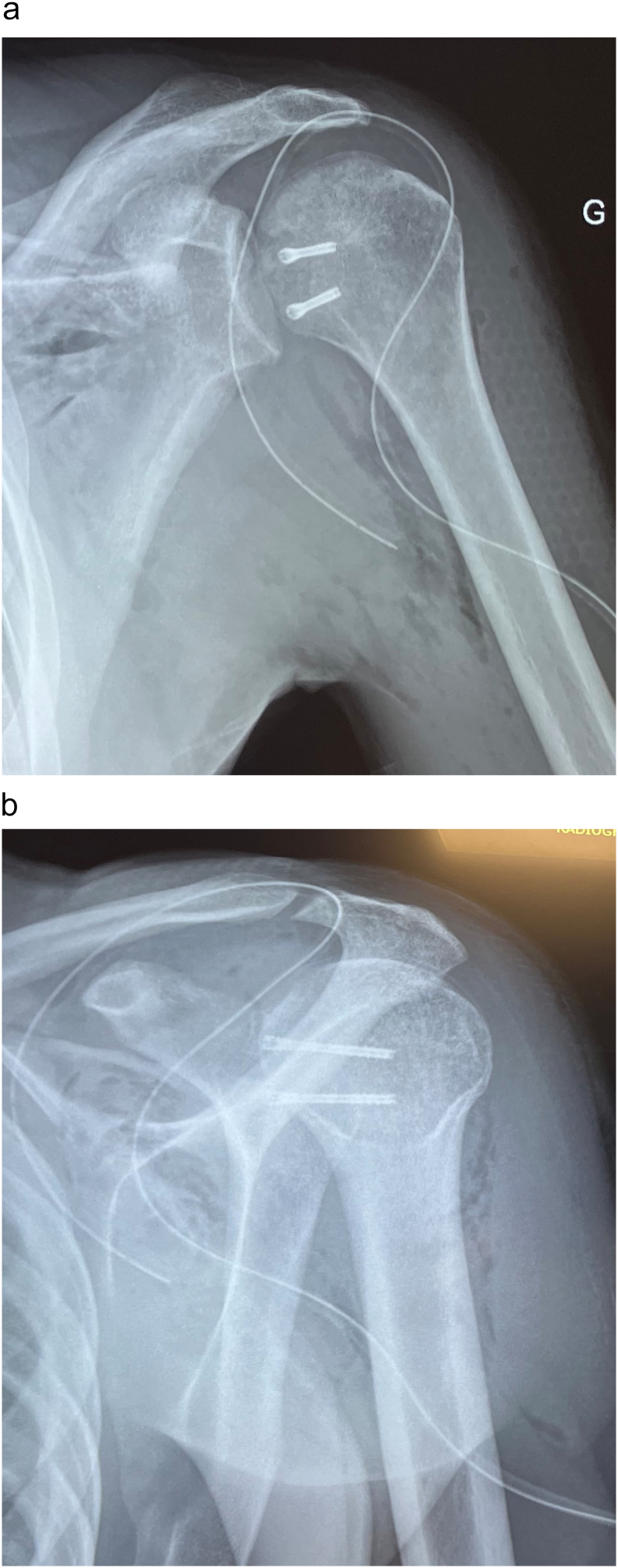
Post operative X-rays.

The graft was inserted in the defect and stabilized with two headless screws. An anchor was placed in the anterior aspect of the humerus head to retension the subscapularis tendon. To ensure a dynamic stabilisation, the long head of the biceps was transferred through the subscapularis after being deinserted at the supraglenoidal notch. The tendon was secured to the anterior rim using a headless anchor (Fig. 3, Fig. 4).

The muscles and skin were then closed using a layered suture technique. Finally, the anesthesiologist and the surgeon collaborated to perform a leak test ensuring the integrity of the thoracic wall and confirming the absence of any air leakage.

The patient was immobilized in a shoulder abduction sling in a neutral rotation position. Rehabilitation began three weeks after surgery, focusing on restoring full range of motion and strengthening the muscles.

**Fig. 5:**
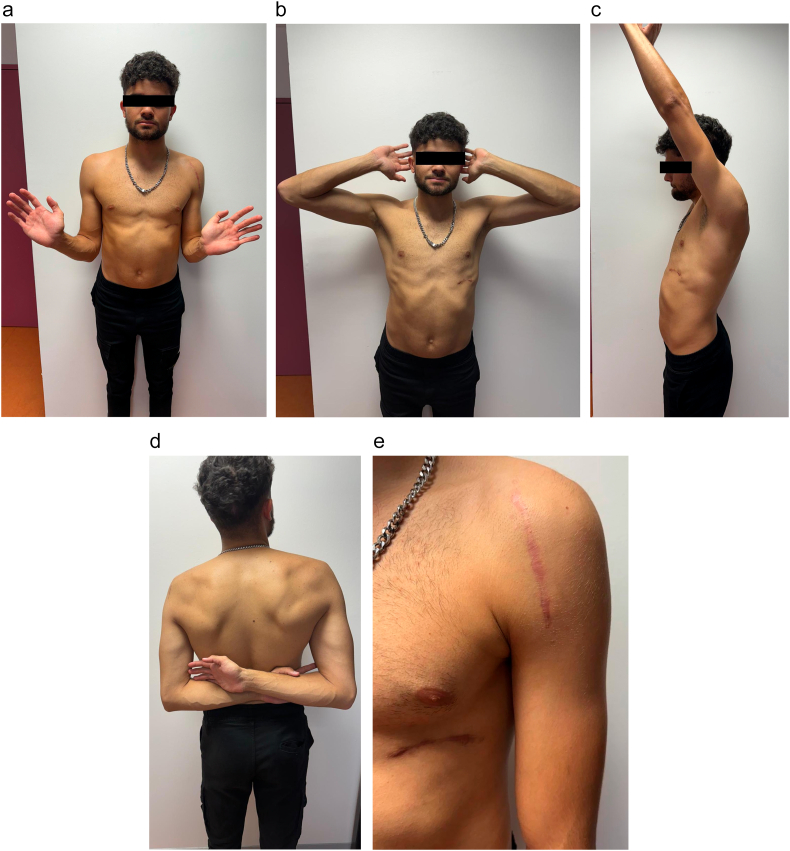
Clinical photographs of the patient during the final follow-up.

The patient was assessed six months after surgery, with a Constant score estimated at 92.5, indicating excellent shoulder function. He successfully resumed his job as a deliveryman and returned to sports activities without any limitations. Physical examination showed a full range of motion, and both Kim and Jerk tests were negative (Fig. 5).

## 3 Discussion

Posterior shoulder dislocation poses a considerable challenge for orthopedic surgeons due to its rarity, ambiguous clinical presentation, and lack of definitive radiographic markers. Its elusive and variable nature often leads to delays in diagnosis and makes treatment complex. It varies from conservative management to total shoulder arthroplasty, depending on several factors outlined in the literature. Key considerations include the size of the reverse Hill–Sachs lesions, dislocation duration, glenoid fossa condition, and the patient's age and overall health [9]. The size of the reverse Hill-Sachs lesion is considered the most significant factor affecting shoulder stability. These lesions are categorized based on the proportion of the articular surface involved: small lesions affect up to 25 %, medium lesions involve 25 % to 50 %, and large lesions encompass more than 50 % of the humeral head [10]. Shoulder instability plays a critical role in determining the treatment plan, with defects exceeding 25 % of the humeral head surface generally necessitating surgical intervention to restore joint stability [11]. Surgical techniques such as the McLaughlin procedure or modified techniques are considered promising alternatives. Lesions involving more than 50 % of the humeral head often require arthroplasty to effectively restore shoulder stability [12]. However, in younger patients, humeral head replacement is usually avoided whenever possible.

Although the modified McLaughlin procedure has shown satisfactory postoperative patient-reported outcomes and range of motion for reverse Hill-Sachs lesions ranging from 25 % to 50 %, concerns remain regarding its effectiveness in maintaining stability for larger defects. Furthermore, some experts have questioned whether subscapularis dysfunction following tendon transfer could affect long-term outcomes and stability in future arthroplasty, particularly due to weakened internal rotation [13]. These concerns may be avoided with costal cartilage grafting.

This technique has been widely used in various surgical fields, particularly in aesthetic and reconstructive procedures. It has been successfully applied in rhinoplasty for nasal framework reconstruction, in mandibular reconstruction for restoring facial contour and function, and in upper limb surgery, including treatments for thumb basal joint arthritis, radiocarpal osteoarthritis, and periscaphoid osteoarthritis. Despite its proven versatility and effectiveness in these areas, its application in reverse Hill-Sachs lesion reconstruction has not been previously reported. This study introduces a novel use of costal cartilage grafting in shoulder surgery, demonstrating its potential as a viable and effective solution for reconstructing humeral head defects in chronic posterior shoulder dislocations. Costal cartilage grafts provide several intrinsic advantages, making them a valuable option for reconstructive procedures. Their strength and abundance ensure a dependable and sufficient supply for grafting. Furthermore, their availability, biocompatibility, and ease of handling enhance their suitability for a wide range of surgical applications [14]. These qualities make costal cartilage a versatile and effective grafting material. This innovative technique provides an anatomical solution that restores joint congruence while preserving shoulder mobility and stability. Compared to other approaches, it ensures excellent biocompatibility and good bone integration, reducing the risk of long-term complications. However, the primary disadvantage reported in the literature is the residual pain that patients may experience; postoperatively [15] which may be mitigated by using minimally invasive techniques [16].

## 4 Conclusion

Our technique, utilizing an osteocartilaginous graft from costal cartilage combined with meticulous soft tissue management, presents a promising approach for reconstructing the humeral head and restoring shoulder stability in chronic posterior shoulder dislocations with large reverse Hill-Sachs lesions.

## CRediT authorship contribution statement

Ala Aloui: Writing original draft.
Jules Cavailhes: Writing, Reviewing and Editing.
Remy Coulomb: Conceptualization, Methodology.
Jeffrey Michaud: Visualization, Investigation.
Pascal Kouyoumdjian: Supervision.
Olivier Mares: Supervision.

## Informed consent

Written informed consent was obtained from all patients and/or families.

## Consent

Written informed consent was obtained from the patient for publication of this case report and accompanying images. A copy of the written consent is available for review by the Editor-in-Chief of this journal on request.

## Ethical approval

Ethical approval was not required.

## Guarantor

Ala Aloui.

## Funding

This research did not receive any specific funding.

## Declaration of competing interest

The authors declare that they have no relevant financial or non-financial interests to report.

## Data Availability

The datasets generated and/or analyzed during the current study are not publicly available due to patient privacy concerns but are available from the corresponding author upon reasonable request.
